# Prevalence and Context of Sunburn Among U.S. Adults — United States, 2024

**DOI:** 10.15585/mmwr.mm7519a2

**Published:** 2026-05-21

**Authors:** Dawn M. Holman, Michael A. Boring, Anne K. Julian, Richard P. Moser, Keegan T. Peterson, Frank M. Perna

**Affiliations:** ^1^Division of Cancer Prevention and Control, National Center for Chronic Disease Prevention and Health Promotion, CDC; ^2^Division of Cancer Control and Population Sciences, National Cancer Institute, Bethesda, Maryland.

**Summary**

**What is already known about this topic?**

Sunburn is a risk factor for skin cancer.

**What is added by this report?**

The 2024 National Health Interview Survey found that an estimated 88.1 million (35.1%) U.S. adults experienced sunburn during the previous year, including 18.8 million (7.5%) who had four or more sunburns. Engaging in water-related activities was the most frequently reported sunburn context (60.6%), followed by exercising (24.7%), drinking alcohol (17.6%), intentional tanning (15.9%), and working at a job (12.9%). Approximately one half (55.1%) of respondents reported that their most recent sunburn occurred despite using sunscreen; education about proper sunscreen use is needed.

**What are the implications for public health practice?**

The findings in this report can guide public health activities to adapt, tailor, and expand existing evidence-based interventions to enhance sun protection, prevent sunburn, and reduce skin cancer risk.

## Abstract

Sunburn is an important risk factor for skin cancer. Understanding sunburn prevalence and the contexts in which sunburn commonly occurs might help guide the development and implementation of sun safety interventions. Data from the 2024 National Health Interview Survey sample adult questionnaire were analyzed to describe the prevalence of one or more and four or more sunburns among U.S. adults during the previous 12 months and the percentages of U.S. adults who experienced their most recent sunburn while they were working at their job; intentionally tanning; exercising; spending time in, on, or near water; using sunscreen; or drinking alcohol. Overall, an estimated 88.1 million (35.1%) U.S. adults had at least one sunburn during the previous 12 months, and 18.8 million (7.5%) had four or more sunburns. Among adults who experienced sunburn during the previous year, spending time in, on, or near the water was the most frequently reported context of their most recent sunburn (60.6%); followed by exercising (24.7%); drinking alcohol (17.6%); intentionally tanning (15.9%); and working at their job (12.9%). Approximately one half (55.1%) of respondents reported that their most recent sunburn occurred despite using sunscreen. These findings can help guide research and activities to adapt, tailor, and expand evidence-based interventions to improve use of sun protection, prevent sunburn, and reduce skin cancer risk.

## Introduction

Skin cancer is the most commonly diagnosed cancer in the United States, with approximately 6 million U.S. adults treated for skin cancer each year and annual treatment costs estimated at $8.9 billion (*1*). Sunburn is the primary modifiable risk factor for skin cancer (*2*,*3*). Even one sunburn at any age increases risk, and a dose-response relationship exists, with more sunburns leading to increasing risk for skin cancer (*2*,*3*). Because of the strong association between a history of sunburn and skin cancer risk, sunburn prevalence can serve as a useful intermediate measure of progress toward reducing skin cancer at a population level (*4*,*5*). Understanding the common contexts in which sunburn occurs is also important for guiding the development and implementation of effective prevention interventions. This report builds on previous research (*6*,*7*) as the first analysis of sunburn context data from a large, nationally representative surveillance survey. This report analyzes data from the 2024 National Health Interview Survey (NHIS) sample adult questionnaire to describe the prevalence of one or more and four or more sunburns among U.S. adults during the previous 12 months and the percentages of U.S. adults who were doing each of the following when they experienced their most recent sunburn: working at their job; intentionally tanning; exercising; spending time in, on, or near water; using sunscreen; or drinking alcohol.

## Methods

### Data Source

NHIS is a cross-sectional household interview survey of the noninstitutionalized U.S. civilian population. In 2024, NHIS conducted 32,629 sample adult interviews with a response rate of 47.9%.

### Survey Measures

The 2024 NHIS sample adult questionnaire (administered to persons aged ≥18 years) included questions about sunburn during the previous 12 months. Respondents were first asked if they had a sunburn during the previous 12 months.* Those who answered affirmatively were asked how many times during the previous 12 months they had experienced sunburn. Respondents reporting four or more sunburns were categorized as having frequent sunburns. Respondents with at least one sunburn during the previous 12 months were also asked to recall their most recent sunburn and report if they were 1) working at their job; 2) trying to get a tan; 3) exercising; 4) spending time in, on, or near the water (e.g., a pool, lake, or ocean); 5) using sunscreen; or 6) drinking alcohol. Respondents could indicate multiple contexts during which sunburn occurred.

Sun sensitivity, an indicator of having skin that sunburns easily, was assessed by asking respondents what would happen to their skin after 1 hour of unprotected sun exposure (*8*). Responses were coded into three categories: sun sensitive,^†^ not sun sensitive,^§^ and do not go out in the sun.^¶^

### Data Analysis

Data were weighted to generate nationally representative estimates for all sample adults and to account for sampling probabilities and nonresponse. Variance estimates were calculated using Taylor series linearization. Unadjusted prevalence of having had at least one sunburn and frequent sunburns during the previous 12 months was calculated overall and among sun-sensitive adults by sex, age group, race and ethnicity, highest level of education attained, family income (quantified as a percentage of the federal poverty level), and U.S. Census Bureau region. NHIS imputed income files were used to create the family income variable. Percentages of adults who reported a context of their most recent sunburn during the previous 12 months were calculated overall and by demographic characteristics; the six contexts were not mutually exclusive. Subgroup percentages were compared with a referent group using design-based t-tests; all differences are significant (with p<0.05 as the level of significance). Because analyses focused on a priori comparisons of subgroups to a prespecified referent category, no adjustments for multiple comparisons were made. The data were analyzed using SAS-callable SUDAAN (version 11.0.3; RTI International) to account for the complex sampling design. This activity was reviewed by CDC, deemed not research, and conducted consistent with applicable federal law and CDC policy.**

## Results

### Sunburn Prevalence

In 2024, approximately 88.1 million (35.1%) U.S. adults, including 68.1 million (54.6%) sun-sensitive adults, reported having had at least one sunburn during the previous 12 months (Table 1). Approximately 18.8 million (7.5% of U.S. adults and 13.2% of sun-sensitive U.S. adults) reported frequent (four or more) sunburns during the previous 12 months (Table 2). Between-group comparisons highlighted differences in sunburn prevalence on the basis of sex, age, race and ethnicity, education, family income, and region (Table 1) (Table 2).

**TABLE 1:** Prevalence of experiencing one or more sunburns during the previous 12 months among adults, by demographic characteristics and sun sensitivity* — National Health Interview Survey, United States, 2024

Characteristic	Total adults					Sun-sensitive adults				
	Sample size	Unweighted no.	Estimated no., millions^†^	% (95% CI)	p-value	Sample size	Unweighted no.	Estimated no., millions^†^	% (95% CI)	p-value
**Total**	**31,603**	**10,494**	**88.1**	**35.1 (34.1–36.0)**	—	**16,169**	**8,207**	**68.1**	**54.6 (53.6–55.7)**	**—**
**Sex**										
Men (Ref)	14,497	4,955	42.9	35.1 (34.0–36.3)	—	6,864	3,713	31.6	56.8 (55.3–58.3)	—
Women	17,101	5,535	45.1	35.0 (33.9–36.1)	0.79	9,302	4,491	36.4	52.9 (51.5–54.2)	0
**Age group, yrs**										
18–29 (Ref)	3,962	1,916	23.0	46.0 (44.0–48.1)	—	1,983	1,430	17.2	71.6 (69.0–74.1)	—
30–44	7,395	3,458	29.0	44.7 (43.2–46.3)	0.28	4,085	2,718	22.5	65.8 (64.0–67.6)	0
45–64	9,610	3,259	26.4	33.6 (32.3–34.9)	0	5,054	2,583	20.7	51.8 (50.1–53.6)	0
≥65	10,591	1,853	9.7	16.8 (16.0–17.7)	0	5,027	1,469	7.7	29.2 (27.7–30.7)	0
**Race and ethnicity**										
Asian, NH	1,803	320	2.5	16.3 (14.5–18.2)	0	454	196	1.5	42.4 (37.2–47.8)	0
Black or African American, NH	3,105	271	2.6	9.1 (7.9–10.4)	0	370	133	1.3	37.8 (31.9–43.9)	0
Hispanic or Latino	4,581	999	9.7	21.5 (20.1–23.0)	0	1,539	630	5.8	40.0 (37.0–43.1)	0
White, NH (Ref)	21,207	8,603	70.7	45.8 (44.7–46.8)	—	13,443	7,047	57.7	57.7 (56.5–58.8)	—
Other races, NH^§^	907	301	2.5	35.8 (31.7–40.1)	0	363	201	1.7	60.2 (53.5–66.4)	0.46
**Highest education level attained**										
Less than high school or GED certificate	2,555	410	4.6	18.2 (16.3–20.2)	0	864	298	3.4	38.8 (35.0–42.8)	0
High school graduate or GED certificate	8,095	2,250	20.1	29.8 (28.4–31.3)	0	3,728	1,725	15.4	51.1 (48.9–53.3)	0
Some college	8,785	3,017	27.4	37.8 (36.4–39.3)	0	4,561	2,295	20.4	56.2 (54.2–58.1)	0.04
College degree (Ref)	12,035	4,790	35.7	42.2 (41.0–43.4)	—	6,974	3,870	28.7	58.6 (57.2–60.0)	—
**Family income (% of FPL)^¶^**										
Poor or near poor (<125)	4,496	951	7.9	23.3 (21.4–25.3)	0	1,730	709	5.8	46.7 (43.2–50.3)	0
Low income (125 to <200)	4,383	1,070	8.6	25.2 (23.5–27.1)	0	1,933	804	6.3	45.6 (42.4–48.8)	0
Middle income (200 to <400)	9,423	3,055	24.8	33.0 (31.6–34.4)	0	4,775	2,370	19	52.7 (50.9–54.6)	0
High income (≥400) (Ref)	13,301	5,418	46.7	43.4 (42.2–44.6)	—	7,731	4,324	36.9	59.3 (58.0–60.7)	—
U.S. Census Bureau region**										
Northeast	4,936	1,613	14.8	34.5 (32.4–36.6)	0.005	2,555	1,288	11.6	54.5 (52.0–57.0)	0.05
Midwest	7,325	2,884	21.8	42.0 (40.0–43.9)	0	4,165	2,291	17.3	59.5 (57.3–61.6)	0
South (Ref)	11,453	3,257	29.8	30.7 (29.3–32.3)	—	5,243	2,481	22.6	51.4 (49.5–53.2)	—
West	7,889	2,740	21.6	36.6 (34.7–38.5)	0	4,206	2,147	16.6	54.9 (52.6–57.2)	0.02

**Abbreviations:** FPL = federal poverty level; GED = general educational development; NH = not Hispanic or Latino; Ref = referent group.

* Respondents were asked to describe what would happen if, after several months of not being in the sun, they went out into the sun without sunscreen or protective clothing for 1 hour. Those who said that they would “get a severe sunburn with blisters,” “have a moderate sunburn with peeling,” or “burn mildly with some or no darkening or tanning” were coded as sun sensitive; those who said they would “turn darker without sunburn” or “nothing would happen to my skin” were coded as not sun sensitive.

^† ^The estimated number of U.S. adults who experienced one or more sunburns during the previous 12 months.

^§^ NH respondents who were American Indian or Alaska Native, or multiple races.

^¶^ Calculated using National Health Interview Survey imputed income files.

** U.S. Census Bureau regions and divisions

**TABLE 2:** Prevalence of experiencing four or more sunburns during the previous 12 months among adults, by demographic characteristics and sun sensitivity* — National Health Interview Survey, United States, 2024

Characteristic	Total adults					Sun-sensitive adults				
	Sample size	Unweighted no.	Estimated no., millions^†^	% (95% CI)	p-value	Sample size	Unweighted no.	Estimated no., millions^†^	% (95% CI)	p-value
**Total**	**31,476**	**2,117**	**18.8**	**7.5 (7.1–8.0)**	**—**	**16,076**	**1,826**	**16.3**	**13.2 (12.4–13.9)**	**—**
**Sex**										
Men (Ref)	14,413	1,075	9.7	8.0 (7.4–8.6)	—	6,801	897	8.2	14.8 (13.7–16.0)	—
Women	17,058	1,040	9.0	7.0 (6.5–7.6)	0.01	9,272	928	8.1	11.9 (11.0–12.8)	0
**Age group, yrs**										
18–29 (Ref)	3,942	566	6.9	13.9 (12.5–15.3)	—	1,967	488	6.1	25.5 (23.1–28.0)	—
30–44	7,361	763	6.2	9.7 (8.8–10.5)	0	4,058	668	5.4	15.8 (14.5–17.2)	0
45–64	9,578	520	4.3	5.5 (5.0–6.1)	0	5,033	444	3.7	9.4 (8.4–10.4)	0
≥65	10,551	265	1.3	2.3 (2.0–2.6)	0	4,998	223	1.1	4.4 (3.8–5.1)	0
**Race and ethnicity**										
Asian, NH	1,801	50	0.4	2.4 (1.7–3.4)	0	454	35	0.3	7.7 (5.4–10.9)	0
Black or African American, NH	3,098	29	0.3	0.9 (0.6–1.3)	0	367	19	0.2	4.8 (2.9–8.0)	0
Hispanic or Latino	4,567	156	1.5	3.4 (2.8–4.0)	0	1,531	107	1.0	6.7 (5.4–8.2)	0
White, NH (Ref)	21,106	1,825	16.2	10.5 (9.9–11.1)	—	13,363	1,618	14.5	14.6 (13.7–15.5)	—
Other races, NH^§^	904	57	0.5	7.0 (5.1–9.6)	0.003	361	47	0.4	14.4 (10.2–19.9)	0.95
**Highest level of education attained**										
Less than high school or GED certificate	2,544	78	1.0	4.0 (3.1–5.3)	0	858	61	0.8	9.1 (6.8–12.0)	0
High school graduate or GED certificate	8,046	430	4.1	6.1 (5.4–6.8)	0	3,696	364	3.5	11.9 (10.5–13.4)	0.005
Some college	8,755	571	5.7	7.9 (7.1–8.7)	0.002	4,538	490	4.9	13.6 (12.2–15.1)	0.34
College degree (Ref)	11,999	1,033	8.0	9.5 (8.8–10.2)	—	6,942	907	7.0	14.4 (13.4–15.5)	—
**Family income (% of FPL)^¶^**										
Poor or near poor (<125)	4,475	187	1.6	4.7 (3.9–5.7)	0	1,718	155	1.3	10.9 (8.9–13.2)	0.003
Low income (125 to <200)	4,367	219	1.9	5.7 (4.8–6.7)	0	1,922	177	1.6	11.5 (9.6–13.7)	0.01
Middle income (200 to <400)	9,385	603	5.1	6.8 (6.2–7.6)	0	4,748	519	4.4	12.3 (11.1–13.7)	0.01
High income (≥400) (Ref)	13,249	1,107	10.1	9.4 (8.7–10.2)	—	7,689	975	9.0	14.5 (13.4–15.6)	—
U.S. Census Bureau region**										
Northeast	4,911	288	2.9	6.7 (5.6–7.9)	0.70	2,537	259	2.6	12.2 (10.2–14.5)	0.78
Midwest	7,293	597	4.7	9.1 (8.3–10.1)	0	4,140	526	4.2	14.7 (13.3–16.1)	0.003
South (Ref)	11,410	639	6.2	6.4 (5.8–7.1)	—	5,212	532	5.2	11.8 (10.7–13.1)	—
West	7,862	593	5.0	8.5 (7.6–9.4)	0	4,187	509	4.3	14.3 (12.9–15.9)	0.01

**Abbreviations:** FPL = federal poverty level; GED = general educational development; NH = not Hispanic or Latino; Ref = referent group.

* Respondents were asked to describe what would happen if, after several months of not being in the sun, they went out into the sun without sunscreen or protective clothing for 1 hour. Those who said that they would “get a severe sunburn with blisters,” “have a moderate sunburn with peeling,” or “burn mildly with some or no darkening or tanning” were coded as sun sensitive; those who said they would “turn darker without sunburn” or “nothing would happen to my skin” were coded as not sun sensitive.

^†^ The estimated number of U.S. adults who experienced four or more sunburns during the previous 12 months.

^§^ Includes NH respondents who were American Indian or Alaska Native or multiple races.

^¶^ Calculated using National Health Interview Survey imputed income files.

** U.S. Census Bureau regions and divisions

### Sunburn Context

Among adults who experienced at least one sunburn during the previous 12 months, 55.1% reported that they experienced their most recent sunburn despite using sunscreen. The most frequently reported sunburn context was spending time in, on, or near water (60.6%), followed by exercising (24.7%), drinking alcohol (17.6%), intentionally tanning (15.9%), and working at their job (12.9%).

**Sex.** Women were significantly more likely than were men to report experiencing a sunburn while spending time in, on, or near water (65.0% versus 55.9%); using sunscreen (64.3% versus 45.3%); and intentionally tanning (21.3% versus 10.3%) (Figure). Men were more likely than were women to report experiencing a sunburn while exercising (27.2% versus 22.4%), drinking alcohol (20.0% versus 15.4%), and working at their job (19.4% versus 6.8%).

**FIGURE:**
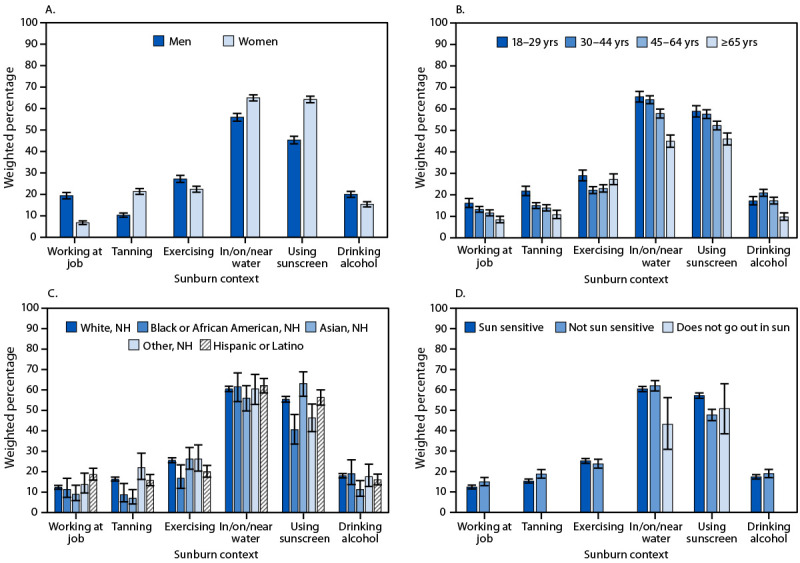
Percentage of adults* whose most recent sunburn occurred while working; tanning; exercising; in, on, or near water; using sunscreen; or drinking alcohol, by sex (A), age group (B), race and ethnicity^†^ (C), and sun sensitivity^§^ (D) — National Health Interview Survey, United States, 2024^¶^ **Abbreviation: **NH = not Hispanic or Latino. * Among persons who reported experiencing any sunburn during the previous 12 months; 95% CIs are shown by error bars. ^†^ NH other includes all NH respondents who self-identified as a race other than Asian, Black or African American, or White or who identified as multiple races. ^§^ Respondents were asked to describe what would happen if, after several months of not being in the sun, they went out into the sun without sunscreen or protective clothing for 1 hour. Those who said that they would “get a severe sunburn with blisters,” “have a moderate sunburn with peeling,” or “burn mildly with some or no darkening or tanning” were coded as sun sensitive; those who said, “turn darker without sunburn” or “nothing would happen to my skin” were coded as not sun sensitive; those who responded to the sun sensitivity question by saying they “do not go out in the sun” were coded as does not go out in the sun. For sun sensitivity (working at their job, tanning, exercising, and drinking alcohol), estimates were suppressed if the minimum effective sample size was <30, the absolute CI width was ≥30 percentage points, or the absolute CI width was 5–30 percentage points, and the relative CI width was >130%. The 95% CIs were calculated using the Korn and Graubard method. ^¶^ Significant differences for subgroup testing: women versus men for all contexts (p<0.001 for each comparison); age 18–29 years versus 30–44 years for working (p = 0.02); 18–29 years versus 45–64 years and ≥65 years for working (p<0.001 for each comparison); 18–29 years versus all other age groups for tanning (p<0.001 for each comparison); 18–29 years versus 30–44 years and 45–64 years for exercising (p<0.001 for each comparison); 18–29 years versus 45–64 years and ≥65 years for water and for sunscreen (p<0.001 for each comparison); 18–29 years versus 30–44 years for alcohol (p = 0.003); 18–29 years versus ≥65 years for alcohol (p<0.001); NH White versus Hispanic or Latino for working (p<0.001); NH White versus NH Black or African American for tanning (p = 0.001); NH White versus NH Asian for tanning (p<0.001); NH White versus NH Black or African American for exercising (p = 0.004); NH White versus Hispanic or Latino for exercising (p<0.001); NH White versus NH Black or African American for sunscreen (p<0.001); NH White versus NH Asian for sunscreen (p = 0.02); NH White versus NH other races for sunscreen (p = 0.009); NH White versus NH Asian for alcohol (p = 0.001); sun sensitive versus not sun sensitive for working (p = 0.02); sun sensitive versus not sun sensitive for tanning (p = 0.004); sun sensitive versus do not go out in the sun for water (p = 0.009); and sun sensitive versus not sun sensitive for sunscreen (p<0.001).

**Age.** Sunburn contexts reported by adults in different age groups were compared with those reported by adults aged 18–29 years. Compared with this group, all other age groups were less likely to report experiencing a sunburn while working at their job or intentionally tanning. Adults aged 30–44 years were less likely to report experiencing a sunburn while exercising (22.2% versus 28.9%) and more likely to report experiencing a sunburn while drinking alcohol (20.9% versus 17.2%) than were adults aged 18–29 years. Those aged 45–64 years were less likely to report experiencing a sunburn while exercising (23.0% versus 28.9%); spending time in, on, or near water (57.8% versus 65.7%); or using sunscreen (52.2% versus 58.9%). Adults aged ≥65 years were less likely to report experiencing a sunburn while spending time in, on, or near water (45.0% versus 65.7%); using sunscreen (46.0% versus 58.9%); or drinking alcohol (9.7% versus 17.2%).

**Race and ethnicity.** All sunburn contexts reported by persons of different racial and ethnic groups were compared with those reported by White adults who were not Hispanic or Latino (non-Hispanic). Non-Hispanic Black or African American adults were less likely to report experiencing a sunburn while intentionally tanning (8.7% versus 16.4%), exercising (16.8% versus 25.6%), and using sunscreen (40.6% versus 55.5%). Non-Hispanic Asian adults were more likely to report experiencing a sunburn while using sunscreen (63.1% versus 55.5%) and less likely to report experiencing a sunburn while intentionally tanning (6.9% versus 16.4%) and drinking alcohol (11.2% versus 18.0%). Non-Hispanic adults of other races^††^ were less likely to report experiencing a sunburn while using sunscreen (46.3% versus 55.5%). Hispanic or Latino (Hispanic) adults were more likely to report experiencing a sunburn while working at their job (18.6% versus 12.4%) and less likely to report experiencing a sunburn while exercising (20.0% versus 25.6%).

**Sun sensitivity.** Compared with sun-sensitive adults, adults who were not sun sensitive were more likely to report experiencing a sunburn while working at their job (15.0% versus 12.4%) and intentionally tanning (18.8% versus 15.3%) and less likely to report experiencing a sunburn while using sunscreen (47.7% versus 57.2%). Adults who reported that they do not go out in the sun were less likely to report experiencing a sunburn while spending time in, on, or near water (43.1%) than were those who were sun sensitive (60.4%).

## Discussion

Each year, approximately 88 million U.S. adults of all races and ethnicities experience at least one sunburn, with approximately 18 million experiencing four or more sunburns. Given the extensive evidence that sunburn increases risk for skin cancer (*2*,*3*), activities to decrease sunburn prevalence might help reduce skin cancer incidence rates over time.

Findings indicate a particularly high sunburn prevalence among adults who are younger, White, sun sensitive, and have a high family income. These demographic groups could be considered when identifying populations of focus for sun safety interventions. Findings regarding the contexts in which adults often experience sunburns can be used to guide future research and intervention approaches. For example, approximately one half of adults who experienced sunburn during the previous year reported that their most recent sunburn occurred while they were using sunscreen. This finding suggests a potential need for interventions to support effective sunscreen use (e.g., use of broad-spectrum^§§^ sunscreen with a sun protection factor [SPF] of ≥15 in combination with protective clothing, applying a sufficient amount [before the application of insect repellent], and frequent reapplication, particularly after swimming or sweating).

Findings regarding specific settings that might pose a high sunburn risk (e.g., outdoor aquatic settings and work spaces) can help to guide the adaptation, tailoring, and expansion of evidence-based interventions designed to influence sun safety knowledge, attitudes, and behaviors while also using environmental approaches (e.g., adequate shade and availability of free sunscreen) to make sun safety easier, particularly in occupational, recreational, and tourism settings. For example, sun safety interventions designed for certain aquatic settings (e.g., the Pool Cool program, a sun safety program for outdoor swimming pools) could be adapted for beaches, lakes, and rivers. Interventions for outdoor workers (e.g., Go Sun Smart) could be expanded, particularly for men, young adults, and Hispanic adults. The results also suggest that adults often experience sunburns while they are engaging in outdoor exercise, tanning, or using alcohol. The high prevalence of sunburn while exercising highlights an opportunity to pair sun safety and physical activity messaging and to promote access to shade (*9*,*10*) in community spaces often used for physical activity (e.g., parks, playgrounds, and sidewalks). Future research could identify strategies to reduce intentional tanning, especially among women, approximately 20% of whom experienced their most recent sunburn while tanning. In addition, future research could examine the potential influence of alcohol use on sunburn risk.

### Limitations

The findings in this report are subject to at least five limitations. First, because the data are cross-sectional, conclusions cannot be drawn regarding causality. Second, because the data are self-reported, they are subject to social desirability and recall bias and might have resulted in an underestimate of number of sunburns. Third, the sunburn context data are limited to respondents’ most recent sunburn and the contexts included on the 2024 NHIS, which likely do not capture all potential contexts in which sunburns might have occurred and might not reflect respondents’ usual sunburn contexts. Fourth, certain respondents selected multiple contexts; however, not enough information was available to determine whether one contextual factor had a larger influence on sunburn occurrence than others, and the different contexts might have influenced one another (e.g., using sunscreen and trying to get a tan while at the beach). Finally, the question about sunscreen use did not record factors that might have affected the effectiveness of the product, such as frequency of application and the type or amount used.

### Implications for Public Health Practice

Each year, approximately one-third of U.S. adults experience at least one sunburn, thereby increasing their skin cancer risk. Several contexts, including outdoor aquatic and occupational settings, outdoor exercise, tanning, and alcohol use, might increase the likelihood of experiencing a sunburn. Sunscreen needs to be used properly and reapplied frequently to prevent sunburn. Measures to adapt, tailor, and expand existing evidence-based sun safety interventions might help prevent sunburns and reduce the risk for skin cancer.

## Footnotes

* Respondents were asked, “During the past 12 months, did you have a sunburn on even a small part of your skin?” If necessary, the interviewer read the following definition of sunburn: “By sunburn, we mean when even a small part of your skin turns red or hurts for 12 hours or more.”

† Respondents were asked, “After several months of not being in the sun, if you then went out into the sun without sunscreen or protective clothing for one hour, which of these would happen to your skin?” Those who said they would “get a severe sunburn with blisters,” “have a moderate sunburn with peeling,” or “burn mildly with some or no darkening or tanning” were coded as sun sensitive.

§ Those who said they would “turn darker without sunburn” or “nothing would happen to my skin” were coded as not sun sensitive.

¶ Those who responded “do not go out in the sun” were coded as does not go out in the sun.

** 45 C.F.R. part 46.102(l)(2), 21 C.F.R. part 56; 42 U.S.C. Sect. 241(d); 5 U.S.C. Sect. 552a; 44 U.S.C. Sect. 3501 et seq.

†† Includes non-Hispanic respondents who were American Indian or Alaska Native or multiple races.

§§ Broad-spectrum sunscreens help protect against ultraviolet A and ultraviolet B radiation, both of which can increase skin cancer risk.
